# First-principles insights on the electronic and optical properties of ZnO@CNT core@shell nanostructure

**DOI:** 10.1038/s41598-018-33991-x

**Published:** 2018-10-18

**Authors:** Yang Shen, Xiaodong Yang, Yue Bian, Kuiying Nie, Songmin Liu, Kun Tang, Rong Zhang, Youdou Zheng, Shulin Gu

**Affiliations:** 1 School of Electronic Science and Engineering, Nanjing University, Nanjing, 210093 China; 2 Institute National Laboratory of Solid State Microstructures and Department of Physics, Nanjing University, Nanjing, 210093 China; 3 Collaborative Innovation Center of Solid-State Lighting and Energy-Saving Electronics, Nanjing University, Nanjing, 210093 China; 4 Collaborative Innovation Center of Advanced Microstructures, Nanjing University, Nanjing, 210093 China; 5 School of Physics and Engineering, Xingyi Normal University for Nationalities, Xingyi, 562400 China

## Abstract

In recent years, various kinds of ZnO-based core@shell nanomaterials have been paid much attention due to their widespread applications in the fields of physics, chemistry and energy conversion. In this work, the electronic and optical properties of a new type of ZnO-based one-dimensional core@shell nanostructure, which is composed of inner ZnO nanowire and outer carbon nanotube (CNT), is calculated based on the first-principles density functional theory (DFT). Calculation results suggest that the ZnO nanowire encapsulated in (9, 9)-CNT is the most stable structure from the view of formation energy. The interaction between the inner ZnO nanowire and the outer (9, 9) CNT belongs to a weak van der Waals type. The complex structure is found to possess metallicity for the outer (9, 9) CNT and maintain the wide band gap nature for the inner ZnO nanowire. Under the different external strains, the charge redistribution between inner ZnO nanowire and outer CNT caused by electron tunneling leads to the shift of Dirac point and the band narrowing of inner ZnO nanowire. The inner ZnO nanowire only has light absorption in the UV region, which is consistent with its optical property originating from its wide bandgap nature.

## Introduction

In the last few years, one dimensional (1D) nanostructures, such as nanowires, nanobelts, nanotubes and so on^1–5^, have attracted much interest due to their superior properties, wide range of potential applications and diverse functionalities^6,7^. Meanwhile, core@shell structures exhibit tunable surface properties and enhanced electronic, optical and catalytic performance which are widely used in diverse electronic and photonic device applications^8,9^. Many efforts have been made towards the synthesis of 1D core@shell nanostructures since the coaxial structure discovered by Suenaga *et al*. in 1997^10^. For example, Guo *et al*. reported the novel electronic and optical properties of TiO_2_-CdS and TiO_2_-Au nanotube arrays, which could lead to high catalytic functions^11^. Lou *et al*. reported the Au@ZrO2 nanorattles used as a stable catalyst for CO oxidation under the circumstance of high temperature^12^. Nie *et al*. also reported the extreme absorption enhancement in ZnTe@ZnO core@shell nanowires by interplay of dielectric resonance and plasmonic bowtie nanoantennas which could greatly improve the efficiency of intermediate band solar cells^13^.

Zinc oxide (ZnO) is a II–VI semiconductor with wide band gap (3.37 eV), large exciton binding energy (60 meV) and excellent thermal stability^14^. ZnO is demonstrated to be a promising nanomaterial used in a broad field of technological applications, including photodetectors, solar cells, photonic crystals, transparent conducting oxides and so on^15–18^. The importance of the materials has accelerated the research progress on the fabrication of ZnO micro/nano-structures. A large number of techniques, including metal organic chemical vapor deposition (MOCVD)^19^, catalytic growth via vapor-liquid-solid (VLS) epitaxial process^20^ and pulsed laser deposition (PLD)^21^ have been developed to create highly oriented arrays of anisotropic ZnO nanostructures. However, low mobility limits their development to some extent.

Carbon nanotubes (CNTs) have been attracted much attention due to their excellent physical, mechanical and chemical properties^22–24^. In general, single wall CNT can be built by curling up single layer of graphene along the 2D lattice vector:

$$\overrightarrow{R}=n{\overrightarrow{R}}_{1}+m{\overrightarrow{R}}_{2},$$

where *n* and *m* represent the chiral indices which are directly related with the helicity and electrical properties of CNTs. There exist the following rules: when *n* − *m* = 3*k* (*k* is non-zero integer), CNTs exhibit metallic behavior while semiconducting property with a band gap for conduction is obtained in the case of *n* − *m* = 3*k* ± 1^25^. (n, 0) and (n, n)-CNTs are called zigzag and armchair nanotubes, respectively, and others are chiral types^26^. The electron mobility of the CNT is high enough, however, low on/off ratio and high subthreshold swings restrict its performance in the fields of device applications.

ZnO@CNT hybrid structure using afore-mentioned two materials has advantages of both materials with high on/off ratio of ZnO and high mobility and ambipolar property of CNT. In recent years, high surface area ZnO@CNT core@shell nanoarrays have been successfully synthesized by a two-step growth process^27^. However, previous studies only focus on the fabrication process and related characterization of ZnO@CNT core@shell nanostructures. To our best knowledge, the experimental or theoretical investigations on the electronic and optical properties of ZnO@CNT core@shell nanostructures which are essential to the performance of the devices are rare. In this context, the electronic and optical properties of complex ZnO@CNT core-shell nanostructure are demonstrated by the first-principles calculation. Calculation results indicate that the ZnO nanowire encapsulated in (9, 9)-CNT is the most stable structure from the point view of formation energy. The interaction between the inner ZnO nanowire and outer (9, 9) CNT belongs to a weak van der Waals type. The complex structure is found to possess metallicity for outer (9, 9) CNT and maintain the wide band gap nature for inner ZnO nanowire. Under the different external strains, the charge redistribution between inner ZnO nanowire and outer CNT caused by electron tunneling leads to the shift of Dirac point and the band narrowing of inner ZnO nanowire. The optical absorption of complex structure can be regarded as a simple accumulation of individual ones except for some small deviations. The superior electronic and optical properties of ZnO@CNT make it good candidate for designing new functional micro/nano-devices.

## Calculation Methods and Models

The first-principles calculations are performed by using the Vienna ab initio Simulation Package (VASP)^28,29^ within the framework of DFT. The electron-ion interactions are described by the projector augmented wave method (PAW)^30^. The van der Waals interactions are taken into consideration by adopting DFT-D2 method^31^. The exchange-correlation energy is treated in the generalized-gradient approximation (GGA)^32^ using the Perdew−Burke−Ernzehof (PBE) functional^33^. Based on the energy test, the kinetic energy cut-off for the planewave expansion is set to 500 eV, which can provide sufficient computational accuracy. For the geometry optimization, the energy convergence criteria for electronic and ionic iterations are 10^−5^ eV and 10^−4^ eV/Å, respectively. Vacuum slabs of 15 Å are added along both the *x* and *y* directions to eliminate the interaction of the neighboring nanotubes, and the axes of the nanowire and nanotube is along the *z* direction. The reciprocal space is meshed at 1 × 1 × 10 for the calculation systems using Monkhorste-Pack^34^ meshes.

**Figure 1:**
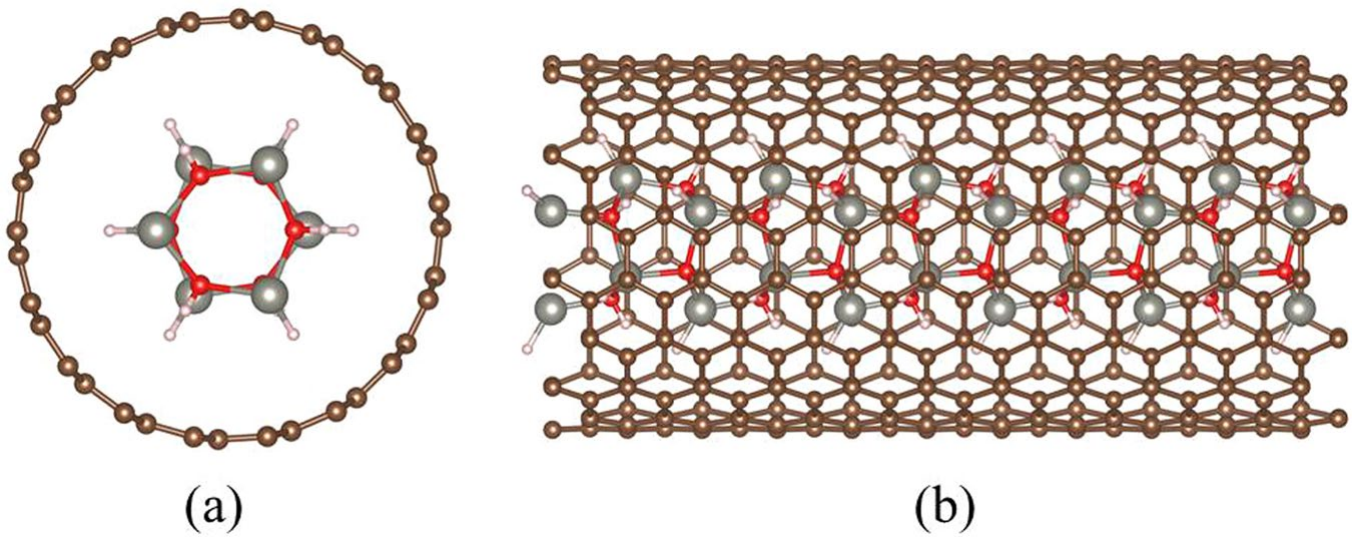
Schematic diagram of ZnO nanowire encapsulated in (9, 9)-CNT (**a**) top view, (**b**) side view. The white, grey, red and brown atoms indicate H atoms, Zn atoms, O atoms in the inner ZnO nanowire and C atoms in the outer CNT respectively.

In order to well encapsulate the inner ZnO nanowire obtained in the experiment^27^, the outer CNT must have enough diameter, and six types of armchair CNTs (n = m = 7–12) are selected to meet the requirements for the encapsulated structure. The chosen (9, 9) CNT is the smallest nanotube among all the possible candidates which could well encapsulate the ZnO nanowire with (11(−)00) surface, and the mismatch between armchair CNT and ZnO is also the lowest. The atomic schematic of ZnO@(9, 9) CNT complex structure is shown in Fig. 1. Due to the single-wall CNTs (SWCNT) are more representative for general study of the core@shell nanostructures, the SWCNT is adopted in our calculations which are different from the multi-wall CNTs (MWCNT) formed in the experiments.

The inner ZnO nanowire has a hexagonal cross-section and exhibits only one hex-atomic ring from overlooking due to the dimension constraints of the CNT outside. The O and Zn atoms are passivated with H atoms. The detailed cell parameters and atomic coordinates of fully optimized ZnO nanowire and ZnO@(n, n) CNT (n = 7–12) are listed in the Supporting Information (Table S1–S7).

## Results and Discussion

**Figure 2:**
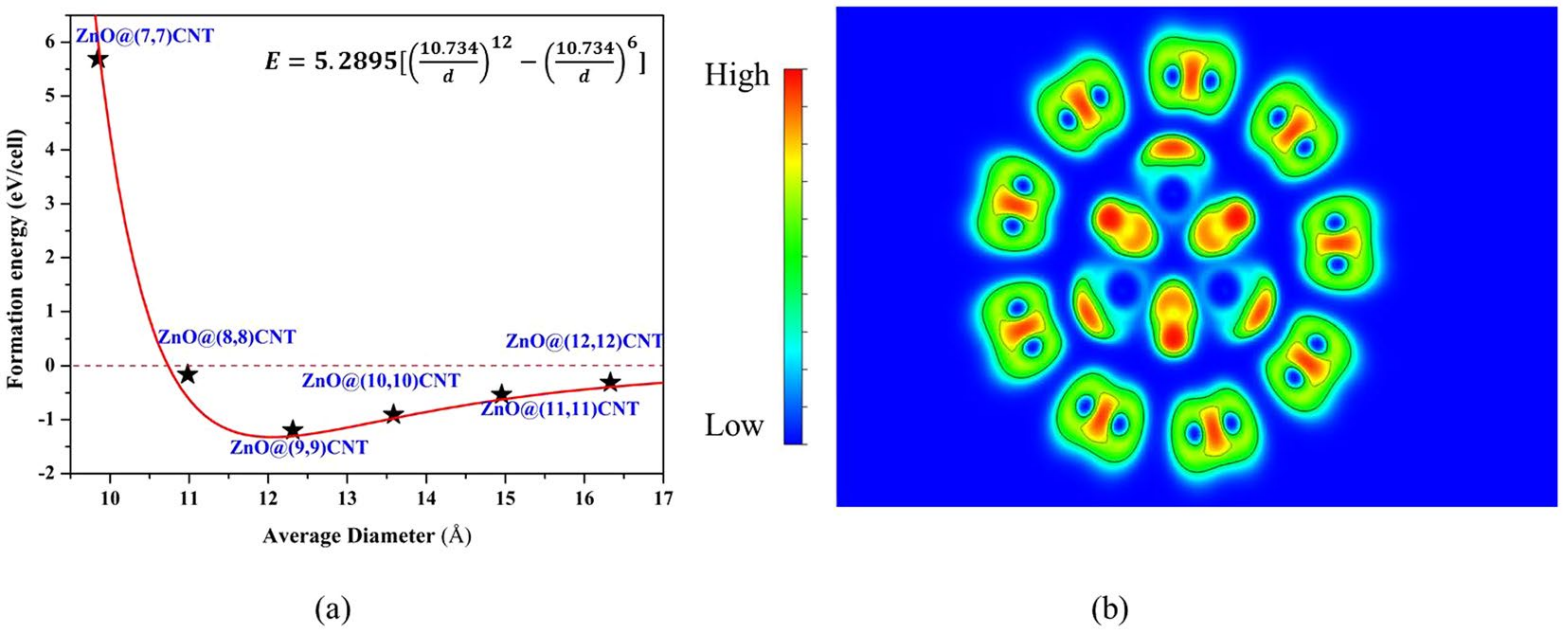
(**a**) The calculated formation energies of different types of ZnO@(n, n) CNT (n = 7–12) complex structure. (**b**) 2D contour plot of electron localization functions on (11(−)00) surface of ZnO@(9, 9) CNT.

To better evaluate the relative stability of the complex ZnO@(n, n) CNT structure, the formation energy (*E*_*f*_) of each encapsulated models were calculated using the following formula^35^:

1 $${E}_{f}={E}_{total}-{E}_{CNT}-{E}_{ZnO}$$

where *E*_*total*_ is the total energy of the complex structure, *E*_*CNT*_ and *E*_*ZnO*_ represent the energy of individual CNT and ZnO, respectively. The calculated formation energies are depicted in Fig. 2(a). If *E*_*f*_ is positive, it means that the encapsulation process is endothermic and the total system is unstable. A negative *E*_*f*_ indicates it is an exothermic chemical process and the stability of the complex structure is enhanced^36^. Obviously, the process of the encapsulated system with too small CNT diameter is endothermic, leading to an unstable structure. The formation energies of ZnO@(n, n) CNT (n = 7–12) system undergo a process that decreases first and then increases. ZnO@(9, 9) CNT owns the least formation energy among all the possible candidates, thus the most stable form is confirmed and used for further investigation. To deeply understand the stability behaviour, we have performed a fitting of the relationship between the formation energy and averaged diameter of ZnO@CNT by using the Lennard-Jones (6–12) potential formula. The Lennard-Jones potential is a mathematically simple model that approximates the interaction between a pair of neutral atoms or molecules. A form of this interatomic potential was first proposed in 1924 by John Lennard-Jones. The most common expression of the L-J potential is

2 $${V}_{LJ}=4\varepsilon [{(\frac{\sigma }{r})}^{12}-{(\frac{\sigma }{r})}^{6}]$$

where ε is the depth of the potential well, σ is the finite distance at which the inter-particle potential is zero, r is the distance between the particles. The fitted result is shown in Fig. 2(a). The relationship between the formation energy and CNT diameter accords with the Lennard-Jones (6–12) potential formula, indicating that the ZnO-CNT interaction in the complex structure belongs to a weak van der Waals type. The chemical bond is always discussed from different points of view, depending upon the chemical and physical aspects or the types of compounds to be examined. Electron localization function (ELF)^37^ measures the extent of spatial localization of the reference electron and provides a method for the mapping of electron pair probability in the multielectronic systems. The type of bonding can be well defined by using ELF plot. As can be seen from Fig. 2(b), the strong covalent bonding between Zn and O atoms and their neighbouring H atoms in the inner ZnO nanowire and the adjacent C atoms in the outer CNT can be inferred from the calculated ELF diagram. No bonding between ZnO nanowire and CNT can be found in the complex structure.

**Figure 3:**
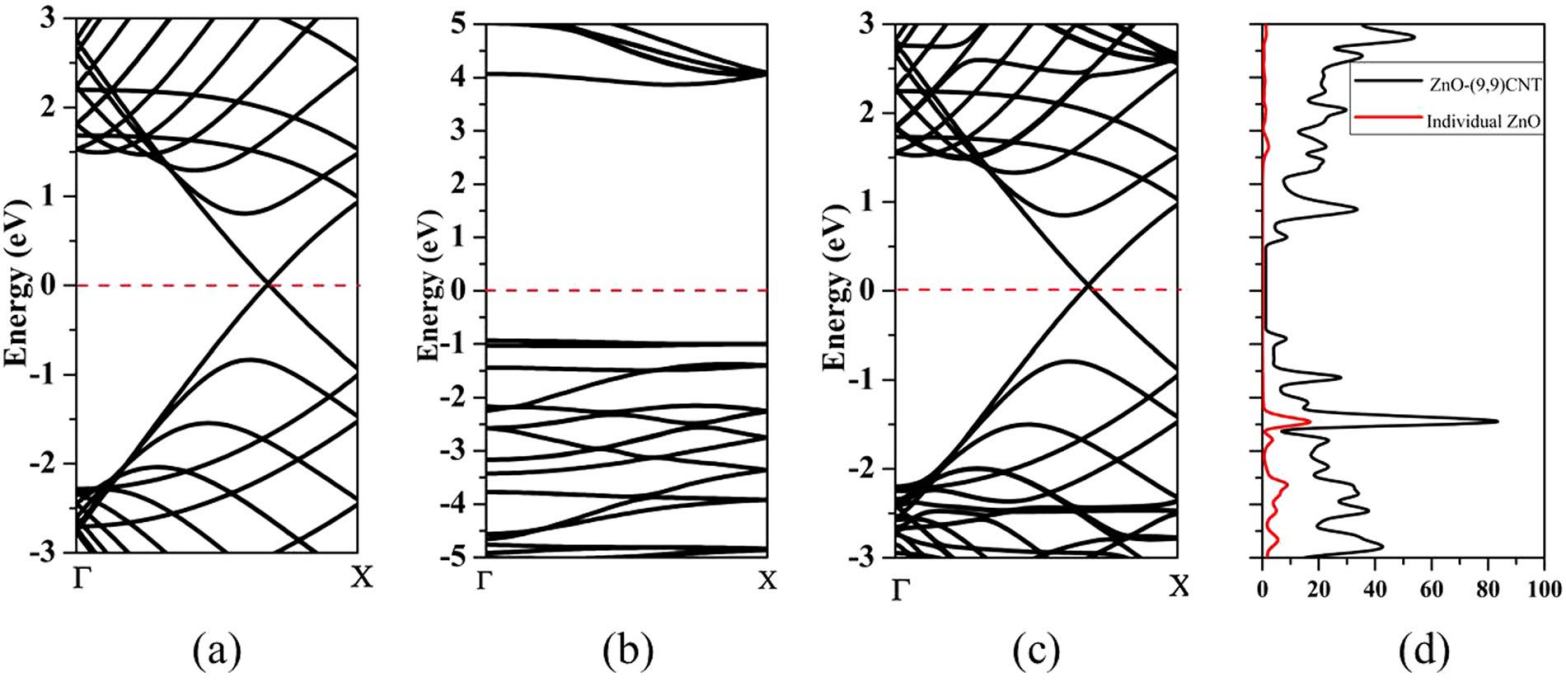
Band structures of (**a**) individual CNT, (**b**) ZnO nanowire and (**c**) ZnO@(9, 9) CNT respectively. (**d**) TDOS of ZnO@(9, 9) CNT and PDOS of individual inner ZnO nanowire. The position of Fermi level is set to energy zero.

Next, electronic structure of the complex structure is calculated and analyzed. The band structures of individual CNT, ZnO and complex structure ZnO@(9, 9) CNT are depicted in Fig. 3(a–c), in which the dotted line represents the Fermi level. The band gap of inner ZnO nanowire is 4.754 eV. The calculated band gap of nanowires is larger than the experimental value (3.37 eV), which is a universal phenomenon due to the well-known quantum size effect^38,39^. The outer armchair CNT is similar to graphene and exhibits the structure of metal Dirac cones from the view of band structure. The complex structure ZnO@(9, 9) CNT maintains the metallic feature of outer CNT (see Fig. 3(c)). The calculated total density of states (TDOS) of ZnO@(9, 9) CNT and partial DOS (PDOS) of individual ZnO nanowire are shown in Fig. 3(d), the inner ZnO nanowire contributes only to the valence band of the electronic structure and basically not participates in conductive of the complex structure.

**Figure 4:**
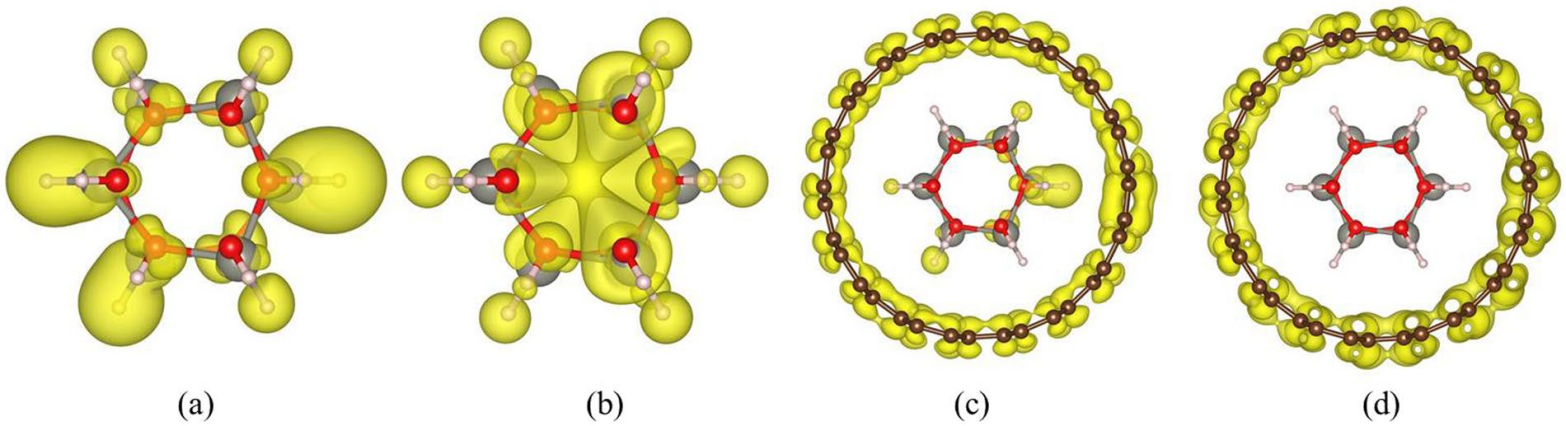
Top views of charge density distributions of (**a**) HOMO and (**b**) LUMO of inner ZnO nanowires, (**c**) HOMO and (**d**) LUMO of complex ZnO@(9, 9) CNT structure.

It is noted that the valence bands are extremely flat near the Fermi level in the ZnO nanowire, as shown in Fig. 3(c). These very flat bands arise from the states located on the surface of the ZnO nanowire which is indicated in Fig. 4. Besides, the vacuum layer between the nanowires breaks the periodicity and leads to local states near Fermi level. Meanwhile, when the H-passivated ZnO nanowire is encapsulated with CNT, the surface states of ZnO nanowire are removed and the band-structure of ZnO@(9, 9) CNT are mainly dominated by outer CNT.

**Figure 5:**
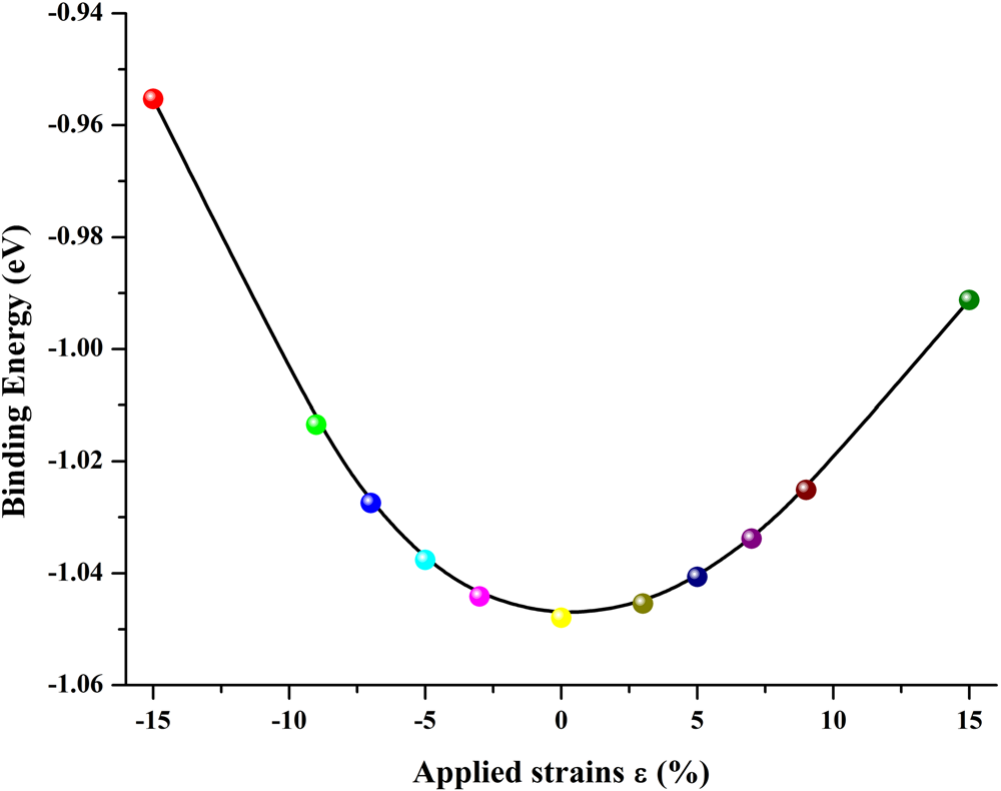
The calculated binding energies of strained ZnO@(9, 9) CNT complex structure.

Next, it should be interesting to examine response of the electronic structure of the complex structure upon the external strain, and specific interaction between the inner and outer in the core-shell structure can take place under the external strains, for example, the core-shell structure can modulate the energy bandgap using the strain effects resulting from the material differences between the core and the shell regions^40–42^. Firstly, the structural stability of strained structure should be evaluated. The binding energies are investigated and depicted in Fig. 5. Obviously, the binding energies are all negative, indicating the ZnO@(9, 9) CNT under different strains will not spontaneous disintegrate and the structure can keep thermally stable. However, the structural stability of the complex structure gets worse as the strain increases. The maximum applied strain is ±15%, which is enough for the investigation of strain effects on the complex core-shell structure.

**Figure 6:**
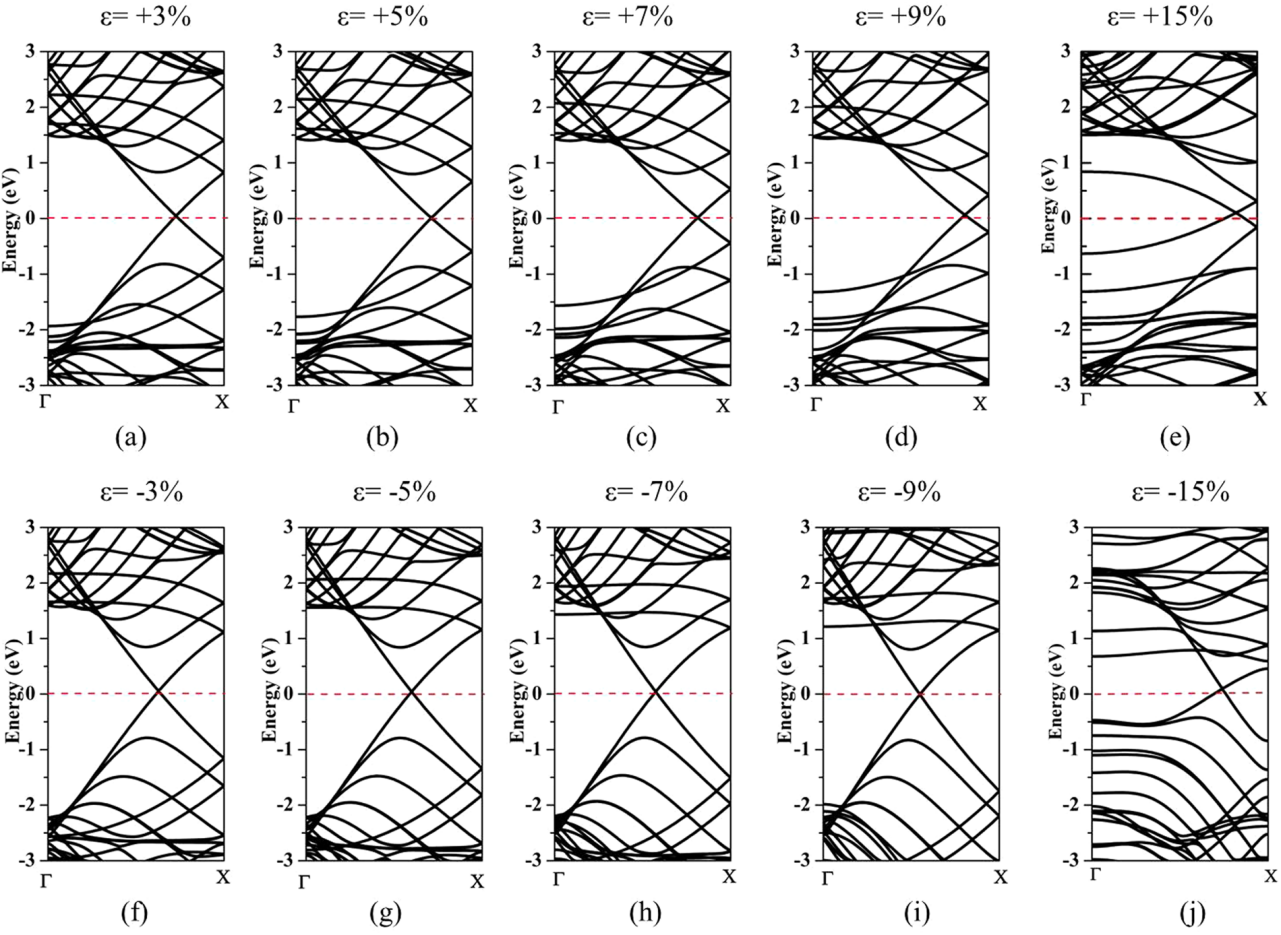
Band structures of ZnO@(9, 9)-CNT under different uniaxial strains (±3–±15%). (**a**–**e**) represent the tensile strain applied along the z axis of the complex structure and (**f**–**j**) represent the compressive strain respectively.

Secondly, the electronics structures of ZnO@(9, 9) CNT under different strains along the *z* axis of the tubes from ±3% to ±15% are calculated and shown in Fig. 6. The negative strains represent the compressive strain on the complex structure while the positive strains indicate the tensile strain respectively. It is noted that the complex structure always keeps metallic properties during the variation of different uniaxial strain. After removing the surface states of inner ZnO nanowire, the highest occupied state at the Γ point of inner ZnO moves relative to the Fermi level regardless of compressive or tensile strains. With the increase of applied strains, the bandgap of inner ZnO narrows down, with a threshold value much smaller than the individual ZnO nanowire represented in Fig. 3. It is noted that the conduction band of inner ZnO almost remains unchanged under the different strains while the valence band shifts upward relative to the Fermi level, leading to the bandgap narrowing. The overall performance of inner ZnO nanowire is related to charge redistribution between outer CNT and inner ZnO in the complex ZnO@(9, 9)-CNT structure. The Dirac point of outer CNT will not fix at the Fermi level and shifts towards the lowest unoccupied molecular orbital (LUMO) of the complex structure when the strains |ε| exceed to 13% (not shown in Fig. 6). The applied strains get the maximum value at ±15% in order to keep the good stability of the complex structure.

**Figure 7:**
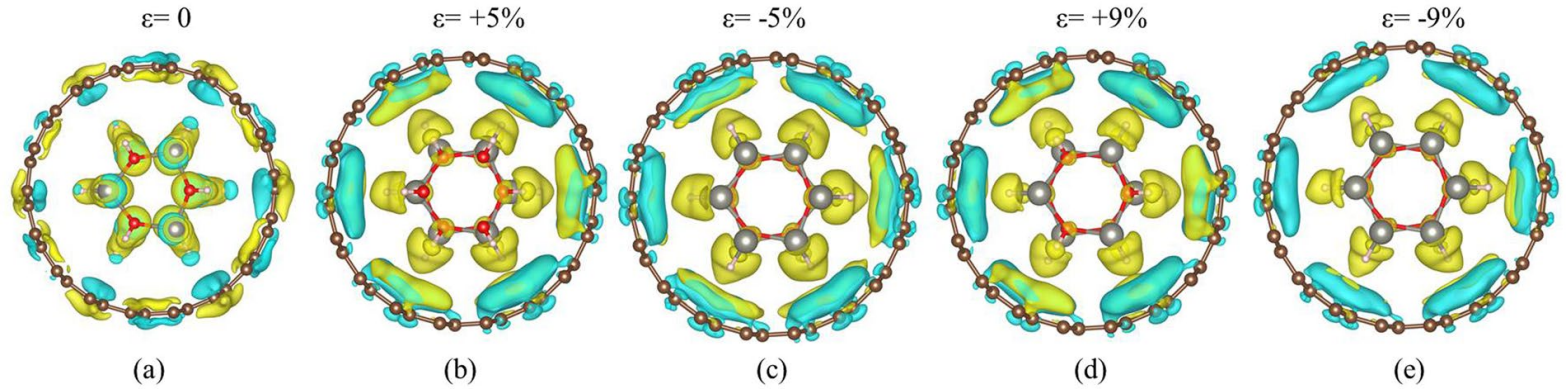
Contour plot of differential charge density of the ZnO@(9, 9)-CNT complex structure under different uniaxial strains (not shown all the applied strains for better display). The yellow and cyan represent the electron depletion and accumulation respectively.

In order to understand the charge redistribution mechanism^43,44^ between inner ZnO nanowire and outer CNT in the ZnO@(9, 9) CNT complex structure, the differential charge density was calculated as shown in Fig. 7. Obviously, there exists electron depletion around the inner ZnO nanowire, which is corresponding to the electron transfer from the inner ZnO to outer CNT. More electrons transfer from ZnO to CNT as the applied strains |ε| increase from 3% to 15%, inducing the Dirac point of outer CNT to shift away from ZnO’s valence band and even exceeds the Fermi level when the applied strains |ε| > 13%.

**Figure 8:**
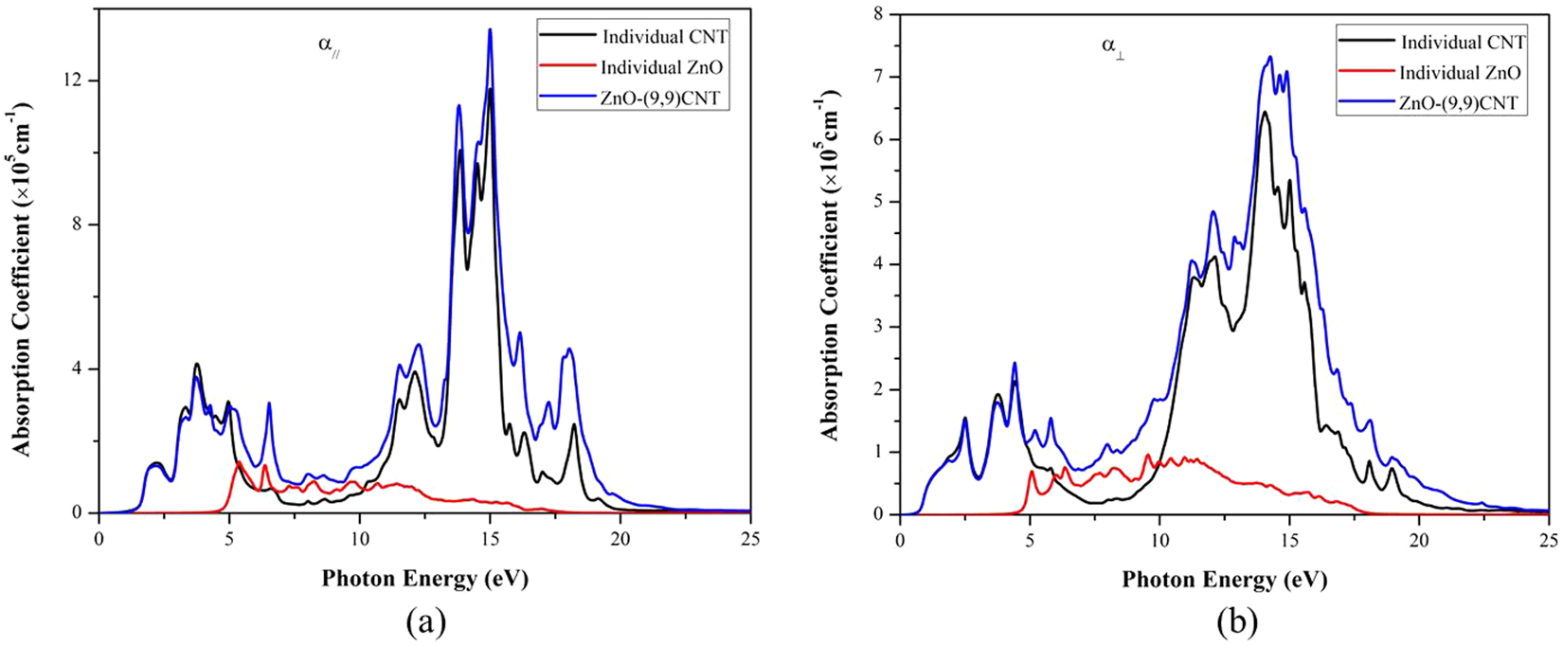
Optical absorption spectrum of individual outer CNT, inner ZnO nanowire and the ZnO@(9, 9)-CNT complex structure. α_⊥_ and α_||_ represent the incident light is perpendicular and parallel to the z axis.

Now we focus on the optical properties of the complex ZnO@(9, 9)-CNT structure. The calculated optical absorption spectrum is depicted in Fig. 8. In the linear response range, the complex dielectric function can be described as^45^:

3 $${\rm{\alpha }}({\rm{\omega }})={{\rm{\varepsilon }}}_{1}({\rm{\omega }})+{{\rm{i}}{\rm{\varepsilon }}}_{2}({\rm{\omega }})$$

where ω is the angular frequency, ε_1_ and ε_2_ represent the real and imaginary part of dielectric function respectively. According to the definitions of direct transition probabilities and Kramers–Kronig dispersion relations^46^, the imaginary and the real parts of the dielectric function can be described, the calculation formulas are as follows^47,48^:

4 $${\varepsilon }_{1}(\omega )=1+\frac{2e}{{\varepsilon }_{0}{m}^{2}}\sum _{V,C}\,\mathop{\int }\limits_{BZ}\,\frac{dK\,{[\alpha \cdot {M}_{V,C}(K)]}^{2}}{2{\pi }^{2}[{E}_{C}(K)-{E}_{V}(K)]/\hslash }\cdot \frac{1}{{[{E}_{C}(K)-{E}_{V}(K)]}^{2}/({\hslash }^{2}-{\omega }^{2})}$$

5 $${\varepsilon }_{2}(\omega )=\frac{\pi }{{\varepsilon }_{0}}{(\frac{e}{m\omega })}^{2}\sum _{V,C}\,\mathop{\int }\limits_{BZ}\,\frac{dK}{2{\pi }^{2}}{[\alpha \cdot {M}_{V,C}(K)]}^{2}\cdot \delta [{E}_{C}(K)-{E}_{V}(K)-\hslash \omega ]$$

where ε_0_ is the vacuum dielectric constant, e and m represent the electron charge and electron mass respectively, ħ is the Planck constant, C and V represent the conduction band and valence band and E_C_(K) and E_V_(K) are the intrinsic level of the conduction band and valence band. BZ represents the first Brillouin zone, K is the electron wave vector, α is the unit vector of the vector potential, M_V,C_ is the unit of transition matrix. The complex refractive index can be expressed as:

6 $${\rm{N}}({\rm{\omega }})={\rm{n}}({\rm{\omega }})+{\rm{ik}}({\rm{\omega }})$$

7 $${\rm{n}}({\rm{\omega }})=\frac{1}{\sqrt{2}}\sqrt{{({{\rm{\varepsilon }}}_{1}^{2}+{{\rm{\varepsilon }}}_{2}^{2})}^{\frac{1}{2}}+{{\rm{\varepsilon }}}_{1}}$$

8 $${\rm{k}}({\rm{\omega }})=\frac{1}{\sqrt{2}}\sqrt{{({{\rm{\varepsilon }}}_{1}^{2}+{{\rm{\varepsilon }}}_{2}^{2})}^{\frac{1}{2}}-{{\rm{\varepsilon }}}_{1}}$$

The absorption coefficient α(E) inferred from the frequency dependent dielectric function can be described as follows^49^:

9 $${\rm{\alpha }}({\rm{E}})\equiv \frac{4{\rm{\pi }}k}{{{\rm{\lambda }}}_{0}}=\frac{4{\rm{\pi }}e}{{\rm{hc}}}\sqrt{\frac{{({{\rm{\varepsilon }}}_{1}^{2}+{{\rm{\varepsilon }}}_{2}^{2})}^{\frac{1}{2}}-{{\rm{\varepsilon }}}_{1}}{2}}$$

It is found that the complex ZnO@(9, 9)-CNT structure can absorb light from ultraviolet (UV) band to near-infrared (NIR) band. The optical absorption of individual ZnO and CNT are also shown in Fig. 8. There exists some small differences in the directions which are perpendicular and parallel to the z axis, however, it does not affect the analysis of optical absorption spectrum. The inner ZnO only has absorption in the UV region, which is consistent with its optical property originating from its wide bandgap nature. The overall optical absorption can be regarded as the superposition of both inner ZnO and outer CNT except for small deviations.

Finally, based on the experience of previous calculation using DFT methods^50,51^, we would like to point out that conventional DFT methods underestimate the band gap of semiconductors while the charge redistribution is usually overestimated^52^. Meanwhile, the underestimation of band gap will influence the calculation accuracy of optical properties, thus the scissor operator correction was used in the calculation to revise the optical results and improve the calculation accuracy. Though DFT methods lack complete accuracy of calculation data, it is good at predicting basic trends and physical mechanisms of calculation systems.

In summary, we performed a systematic theoretical investigation on the electronic and optical properties of the complex ZnO@(9, 9)-CNT core-shell structure. Calculation results indicate that the ZnO nanowire encapsulated in (9, 9)-CNT is the most stable structure from the view of formation energy. The interaction between the inner ZnO nanowire and outer (9, 9)-CNT nanostructure belongs to weak van der Waals. The complex structure is found to possess metallicity for outer (9, 9) CNT and maintain the wide band gap nature for inner ZnO nanowire. Under the different external strain, the charge redistribution between inner ZnO nanowire and outer CNT caused by electron tunneling leads to the shift of Dirac point and the band narrowing of inner ZnO nanowire. The optical absorption of complex structure can be regarded as a simple accumulation of individual ones except for some small deviations. By analysing the electronic and optical properties, we come to conclusions that the complex ZnO@CNT structure could have many potential applications in future nano-optoelectronic devices.

## Electronic supplementary material

Supporting Information
