# Supplementary material for: First-principles insights on the electronic and optical properties of ZnO@CNT core@shell nanostructure
Source: Sci Rep. 2018 Oct 18;8:15464. doi: 10.1038/s41598-018-33991-x (PMC6194084; doi:10.1038/s41598-018-33991-x)
Supplement: Supplementary file 1 — Supporting Information [file 41598_2018_33991_MOESM1_ESM.docx]

**First-principles insights on the electronic and optical properties of ZnO@CNT core@shell nanostructure**

Yang Shen^1,3^, Xiaodong Yang^2^, Yue Bian^1,3^, Kuiying Nie^1,5^, Songmin Liu^1,3,*^,

Kun Tang ^1,3,*^, Rong Zhang^1,3,4^, Youdou Zheng^1^, Shulin Gu^1,3,*^

^1^ School of Electronic Science and Engineering, Nanjing University, Nanjing 210093, China

^2^Institute National Laboratory of Solid State Microstructures and Department of Physics, Nanjing University, Nanjing 210093, China

^3^Collaborative Innovation Center of Solid-State Lighting and Energy-Saving Electronics, Nanjing University, Nanjing 210093, China

*^4^Collaborative Innovation Center of Advanced Microstructures, Nanjing University, Nanjing 210093, China9*

^5^School of Physics and Engineering, Xingyi Normal University for Nationalities, Xingyi 562400, China

-----------------------

**Correspondence and requests for materials should be addressed to S.M. Liu (liusongmin@nju.edu.cn), K. Tang (ktang@nju.edu.cn) and S.L. Gu (slgu@nju.edu.cn)*

**Table S1.** The fully optimized cell parameters and atomic coordinates of individual ZnO nanowire.

| **Cell parameters** | **Atom** | ***x*** | ***y*** | ***z*** |
| --- | --- | --- | --- | --- |
| ***a* = 28.032864 Å**  ***b* = 28.020846 Å**  ***c* = 5.005124 Å** | **H1** | **0.545258** | **0.534118** | **0.346854** |
|  | **H2** | **0.560319** | **0.560051** | **0.69093** |
|  | **H3** | **0.448941** | **0.533886** | **0.845472** |
|  | **H4** | **0.433637** | **0.560297** | **0.193249** |
|  | **H5** | **0.371223** | **0.450006** | **0.68972** |
|  | **H6** | **0.401463** | **0.450637** | **0.344452** |
|  | **H7** | **0.449473** | **0.367192** | **0.84538** |
|  | **H8** | **0.433429** | **0.341591** | **0.191679** |
|  | **H9** | **0.545478** | **0.367349** | **0.347282** |
|  | **H10** | **0.561275** | **0.342066** | **0.68975** |
|  | **H11** | **0.593234** | **0.450978** | **0.847239** |
|  | **H12** | **0.62285** | **0.450521** | **0.19051** |
|  | **O1** | **0.432043** | **0.450755** | **0.246986** |
|  | **O2** | **0.464809** | **0.39366** | **0.747823** |
|  | **O3** | **0.52999** | **0.39365** | **0.248422** |
|  | **O4** | **0.464378** | **0.507464** | **0.747931** |
|  | **O5** | **0.529876** | **0.507729** | **0.248396** |
|  | **O6** | **0.562667** | **0.450931** | **0.748844** |
|  | **Zn1** | **0.419342** | **0.450435** | **0.846838** |
|  | **Zn2** | **0.4583** | **0.382932** | **0.347449** |
|  | **Zn3** | **0.536567** | **0.383125** | **0.848596** |
|  | **Zn4** | **0.458088** | **0.51853** | **0.347518** |
|  | **Zn5** | **0.536133** | **0.51855** | **0.848444** |
|  | **Zn6** | **0.574954** | **0.450755** | **0.349113** |

**Table S2.** The fully optimized cell parameters and atomic coordinates of ZnO@(7,7) CNT.

| **Cell parameters** | **Atom** | ***x*** | ***y*** | ***z*** |
| --- | --- | --- | --- | --- |
| **a = 25.243196 Å**  **b = 25.189123 Å**  **c = 4.936613 Å** | **H1** | **0.543766** | **0.579481** | **0.394225** |
|  | **H2** | **0.552711** | **0.592823** | **0.63749** |
|  | **H3** | **0.441821** | **0.579308** | **0.894505** |
|  | **H4** | **0.433236** | **0.592839** | **0.137493** |
|  | **H5** | **0.37647** | **0.489146** | **0.626481** |
|  | **H6** | **0.392901** | **0.491325** | **0.385701** |
|  | **H7** | **0.443488** | **0.405372** | **0.884398** |
|  | **H8** | **0.433852** | **0.391281** | **0.116222** |
|  | **H9** | **0.542972** | **0.405786** | **0.385766** |
|  | **H10** | **0.552877** | **0.391603** | **0.615931** |
|  | **H11** | **0.59306** | **0.49177** | **0.887975** |
|  | **H12** | **0.609803** | **0.490127** | **0.126544** |
|  | **C1** | **0.686394** | **0.509897** | **0.59689** |
|  | **C2** | **0.600936** | **0.656383** | **0.594599** |
|  | **C3** | **0.670863** | **0.565653** | **0.596138** |
|  | **C4** | **0.658799** | **0.59193** | **0.846159** |
|  | **C5** | **0.54767** | **0.679959** | **0.593882** |
|  | **C6** | **0.51894** | **0.684123** | **0.84353** |
|  | **C7** | **0.623614** | **0.63797** | **0.844814** |
|  | **C8** | **0.433301** | **0.676111** | **0.593677** |
|  | **C9** | **0.461127** | **0.683176** | **0.844401** |
|  | **C10** | **0.311281** | **0.560712** | **0.595482** |
|  | **C11** | **0.381323** | **0.651034** | **0.594064** |
|  | **C12** | **0.358978** | **0.633044** | **0.84489** |
|  | **C13** | **0.297638** | **0.504189** | **0.596548** |
|  | **C14** | **0.297894** | **0.474974** | **0.846927** |
|  | **C15** | **0.324809** | **0.586374** | **0.845044** |
|  | **C16** | **0.327983** | **0.393895** | **0.597857** |
|  | **C17** | **0.313756** | **0.419106** | **0.847836** |
|  | **C18** | **0.467265** | **0.301463** | **0.598856** |
|  | **C19** | **0.363659** | **0.348302** | **0.59839** |
|  | **C20** | **0.385963** | **0.330128** | **0.848684** |
|  | **C21** | **0.525071** | **0.302073** | **0.59916** |
|  | **C22** | **0.553597** | **0.307534** | **0.849334** |
|  | **C23** | **0.438955** | **0.306965** | **0.849019** |
|  | **C24** | **0.62789** | **0.351779** | **0.599045** |
|  | **C25** | **0.605982** | **0.332561** | **0.849242** |
|  | **C26** | **0.662127** | **0.398498** | **0.59823** |
|  | **C27** | **0.67377** | **0.425004** | **0.848145** |
|  | **C28** | **0.687458** | **0.481208** | **0.847478** |
|  | **C29** | **0.688088** | **0.510238** | **0.097193** |
|  | **C30** | **0.599875** | **0.654484** | **0.094399** |
|  | **C31** | **0.67252** | **0.56624** | **0.096073** |
|  | **C32** | **0.658243** | **0.591492** | **0.34564** |
|  | **C33** | **0.547003** | **0.677706** | **0.093754** |
|  | **C34** | **0.518942** | **0.683944** | **0.344408** |
|  | **C35** | **0.622738** | **0.637174** | **0.34523** |
|  | **C36** | **0.432507** | **0.678416** | **0.093724** |
|  | **C37** | **0.461123** | **0.683489** | **0.34343** |
|  | **C38** | **0.31305** | **0.560148** | **0.095519** |
|  | **C39** | **0.380074** | **0.652997** | **0.094231** |
|  | **C40** | **0.358209** | **0.633692** | **0.344359** |
|  | **C41** | **0.299373** | **0.503925** | **0.096255** |
|  | **C42** | **0.29928** | **0.475185** | **0.346799** |
|  | **C43** | **0.324394** | **0.586692** | **0.345699** |
|  | **C44** | **0.327078** | **0.393174** | **0.09762** |
|  | **C45** | **0.314787** | **0.419421** | **0.347446** |
|  | **C46** | **0.467222** | **0.301406** | **0.099073** |
|  | **C47** | **0.362682** | **0.347393** | **0.098615** |
|  | **C48** | **0.385259** | **0.328969** | **0.348918** |
|  | **C49** | **0.525019** | **0.302368** | **0.098919** |
|  | **C50** | **0.55316** | **0.308641** | **0.349163** |
|  | **C51** | **0.438559** | **0.305905** | **0.349166** |
|  | **C52** | **0.626955** | **0.352563** | **0.098807** |
|  | **C53** | **0.605255** | **0.333707** | **0.349031** |
|  | **C54** | **0.661365** | **0.399072** | **0.098439** |
|  | **C55** | **0.674873** | **0.424682** | **0.348423** |
|  | **C56** | **0.688884** | **0.481017** | **0.347577** |
|  | **O1** | **0.422923** | **0.492098** | **0.248823** |
|  | **O2** | **0.45875** | **0.430677** | **0.742581** |
|  | **O3** | **0.527779** | **0.431041** | **0.242957** |
|  | **O4** | **0.457318** | **0.553654** | **0.756863** |
|  | **O5** | **0.528377** | **0.553723** | **0.256969** |
|  | **O6** | **0.563201** | **0.49225** | **0.749576** |
|  | **Zn1** | **0.413269** | **0.49152** | **0.868417** |
|  | **Zn2** | **0.452922** | **0.422141** | **0.362929** |
|  | **Zn3** | **0.533563** | **0.422214** | **0.86341** |
|  | **Zn4** | **0.452951** | **0.56141** | **0.376544** |
|  | **Zn5** | **0.532713** | **0.561526** | **0.876537** |
|  | **Zn6** | **0.572996** | **0.491942** | **0.369216** |

**Table S3.** The fully optimized cell parameters and atomic coordinates of ZnO@(8,8) CNT.

| **Cell parameters** | **Atom** | ***x*** | ***y*** | ***z*** |
| --- | --- | --- | --- | --- |
| **a = 26.049308 Å**  **b = 26.134926 Å**  **c = 4.931443 Å** | **H1** | **0.558641** | **0.568296** | **0.371017** |
|  | **H2** | **0.571132** | **0.590194** | **0.652725** |
|  | **H3** | **0.456516** | **0.568094** | **0.870791** |
|  | **H4** | **0.443697** | **0.590215** | **0.157013** |
|  | **H5** | **0.382018** | **0.479869** | **0.644992** |
|  | **H6** | **0.406586** | **0.480248** | **0.36834** |
|  | **H7** | **0.456779** | **0.392862** | **0.870902** |
|  | **H8** | **0.443181** | **0.373068** | **0.148457** |
|  | **H9** | **0.558639** | **0.393119** | **0.372175** |
|  | **H10** | **0.572043** | **0.373416** | **0.651046** |
|  | **H11** | **0.60871** | **0.480966** | **0.869614** |
|  | **H12** | **0.632918** | **0.480916** | **0.145304** |
|  | **C1** | **0.706841** | **0.545804** | **0.598761** |
|  | **C2** | **0.683399** | **0.59559** | **0.598718** |
|  | **C3** | **0.667885** | **0.618184** | **0.848439** |
|  | **C4** | **0.7137** | **0.519392** | **0.849279** |
|  | **C5** | **0.604925** | **0.671004** | **0.597889** |
|  | **C6** | **0.628357** | **0.656555** | **0.847869** |
|  | **C7** | **0.443695** | **0.683485** | **0.596315** |
|  | **C8** | **0.552651** | **0.688865** | **0.597104** |
|  | **C9** | **0.525378** | **0.692161** | **0.846641** |
|  | **C10** | **0.393485** | **0.6607** | **0.596483** |
|  | **C11** | **0.371448** | **0.6446** | **0.847091** |
|  | **C12** | **0.470259** | **0.68996** | **0.846755** |
|  | **C13** | **0.320814** | **0.579847** | **0.597456** |
|  | **C14** | **0.335024** | **0.603245** | **0.847325** |
|  | **C15** | **0.306645** | **0.419628** | **0.598556** |
|  | **C16** | **0.301447** | **0.528281** | **0.598226** |
|  | **C17** | **0.296789** | **0.501177** | **0.848133** |
|  | **C18** | **0.330223** | **0.369906** | **0.598128** |
|  | **C19** | **0.346244** | **0.347747** | **0.84831** |
|  | **C20** | **0.299467** | **0.446118** | **0.848343** |
|  | **C21** | **0.409405** | **0.295671** | **0.597445** |
|  | **C22** | **0.385986** | **0.309642** | **0.848115** |
|  | **C23** | **0.570876** | **0.28114** | **0.59724** |
|  | **C24** | **0.461579** | **0.2779** | **0.597139** |
|  | **C25** | **0.48865** | **0.273764** | **0.847449** |
|  | **C26** | **0.621443** | **0.303725** | **0.597642** |
|  | **C27** | **0.643365** | **0.320469** | **0.847475** |
|  | **C28** | **0.54383** | **0.275859** | **0.846852** |
|  | **C29** | **0.693245** | **0.385912** | **0.598318** |
|  | **C30** | **0.679568** | **0.362127** | **0.848128** |
|  | **C31** | **0.71246** | **0.437529** | **0.59852** |
|  | **C32** | **0.716753** | **0.464454** | **0.849162** |
|  | **C33** | **0.707132** | **0.545927** | **0.099154** |
|  | **C34** | **0.683537** | **0.595639** | **0.09851** |
|  | **C35** | **0.66756** | **0.617834** | **0.348473** |
|  | **C36** | **0.714483** | **0.519477** | **0.349052** |
|  | **C37** | **0.604486** | **0.670008** | **0.09743** |
|  | **C38** | **0.62784** | **0.655932** | **0.348161** |
|  | **C39** | **0.44333** | **0.684513** | **0.096576** |
|  | **C40** | **0.55242** | **0.688043** | **0.09692** |
|  | **C41** | **0.525395** | **0.692215** | **0.347026** |
|  | **C42** | **0.392914** | **0.661737** | **0.096868** |
|  | **C43** | **0.371034** | **0.645011** | **0.346681** |
|  | **C44** | **0.470279** | **0.690052** | **0.346309** |
|  | **C45** | **0.321125** | **0.579717** | **0.097509** |
|  | **C46** | **0.334832** | **0.603442** | **0.347321** |
|  | **C47** | **0.307032** | **0.419796** | **0.09823** |
|  | **C48** | **0.302007** | **0.52808** | **0.097769** |
|  | **C49** | **0.297667** | **0.501174** | **0.348441** |
|  | **C50** | **0.330428** | **0.37** | **0.098376** |
|  | **C51** | **0.345914** | **0.347366** | **0.348156** |
|  | **C52** | **0.300305** | **0.446234** | **0.348586** |
|  | **C53** | **0.408885** | **0.294443** | **0.097878** |
|  | **C54** | **0.385428** | **0.308938** | **0.34771** |
|  | **C55** | **0.570428** | **0.282447** | **0.096987** |
|  | **C56** | **0.46129** | **0.276827** | **0.097326** |
|  | **C57** | **0.488652** | **0.273794** | **0.346999** |
|  | **C58** | **0.620773** | **0.304978** | **0.097294** |
|  | **C59** | **0.642885** | **0.320989** | **0.348009** |
|  | **C60** | **0.543829** | **0.275998** | **0.347431** |
|  | **C61** | **0.693678** | **0.385743** | **0.098245** |
|  | **C62** | **0.67935** | **0.362362** | **0.348164** |
|  | **C63** | **0.713216** | **0.437301** | **0.098988** |
|  | **C64** | **0.717727** | **0.464443** | **0.348905** |
|  | **O1** | **0.43771** | **0.48068** | **0.249747** |
|  | **O2** | **0.472655** | **0.419455** | **0.751494** |
|  | **O3** | **0.542728** | **0.419643** | **0.252806** |
|  | **O4** | **0.472357** | **0.54134** | **0.75361** |
|  | **O5** | **0.542821** | **0.541588** | **0.252658** |
|  | **O6** | **0.577619** | **0.48113** | **0.750503** |
|  | **Zn1** | **0.425137** | **0.480172** | **0.858072** |
|  | **Zn2** | **0.466517** | **0.409265** | **0.359695** |
|  | **Zn3** | **0.548857** | **0.4097** | **0.861073** |
|  | **Zn4** | **0.466349** | **0.551925** | **0.360334** |
|  | **Zn5** | **0.548918** | **0.552254** | **0.860006** |
|  | **Zn6** | **0.590038** | **0.480653** | **0.35912** |

**Table S4**. The fully optimized cell parameters and atomic coordinates of ZnO@(9,9) CNT.

| **Cell parameters** | **Atom** | ***x*** | ***y*** | ***z*** |
| --- | --- | --- | --- | --- |
| **a = 28.084770 Å**  **b = 28.078119 Å**  **c = 4.930910 Å** | **H1** | **0.545416** | **0.534223** | **0.347522** |
|  | **H2** | **0.560216** | **0.55976** | **0.689469** |
|  | **H3** | **0.448918** | **0.533995** | **0.846223** |
|  | **H4** | **0.433673** | **0.560178** | **0.192325** |
|  | **H5** | **0.371356** | **0.44996** | **0.688277** |
|  | **H6** | **0.401425** | **0.450631** | **0.345056** |
|  | **H7** | **0.449503** | **0.367028** | **0.846029** |
|  | **H8** | **0.433462** | **0.341846** | **0.190683** |
|  | **H9** | **0.545613** | **0.367155** | **0.348026** |
|  | **H10** | **0.561154** | **0.342436** | **0.688133** |
|  | **H11** | **0.593426** | **0.450976** | **0.847878** |
|  | **H12** | **0.622395** | **0.450479** | **0.18917** |
|  | **C1** | **0.713471** | **0.453445** | **0.606816** |
|  | **C2** | **0.711755** | **0.478632** | **0.857128** |
|  | **C3** | **0.688636** | **0.551067** | **0.606963** |
|  | **C4** | **0.699019** | **0.527996** | **0.856938** |
|  | **C5** | **0.57957** | **0.653583** | **0.606361** |
|  | **C6** | **0.660418** | **0.593526** | **0.606871** |
|  | **C7** | **0.643192** | **0.612007** | **0.856641** |
|  | **C8** | **0.5305** | **0.667324** | **0.606139** |
|  | **C9** | **0.505348** | **0.669829** | **0.856018** |
|  | **C10** | **0.602289** | **0.642469** | **0.856393** |
|  | **C11** | **0.429938** | **0.660625** | **0.606025** |
|  | **C12** | **0.454481** | **0.666515** | **0.856107** |
|  | **C13** | **0.310833** | **0.570299** | **0.605791** |
|  | **C14** | **0.383182** | **0.640342** | **0.606064** |
|  | **C15** | **0.362227** | **0.626284** | **0.856082** |
|  | **C16** | **0.288231** | **0.52461** | **0.605599** |
|  | **C17** | **0.280942** | **0.500394** | **0.85549** |
|  | **C18** | **0.325671** | **0.590757** | **0.855928** |
|  | **C19** | **0.276857** | **0.424508** | **0.605417** |
|  | **C20** | **0.275148** | **0.449732** | **0.855405** |
|  | **C21** | **0.345318** | **0.291191** | **0.605124** |
|  | **C22** | **0.289314** | **0.375052** | **0.605281** |
|  | **C23** | **0.299661** | **0.35196** | **0.855154** |
|  | **C24** | **0.386348** | **0.260882** | **0.605088** |
|  | **C25** | **0.409015** | **0.249792** | **0.85539** |
|  | **C26** | **0.327921** | **0.309512** | **0.855086** |
|  | **C27** | **0.483285** | **0.23338** | **0.605565** |
|  | **C28** | **0.458127** | **0.235992** | **0.855614** |
|  | **C29** | **0.626628** | **0.276603** | **0.606248** |
|  | **C30** | **0.534178** | **0.236555** | **0.605934** |
|  | **C31** | **0.558835** | **0.242255** | **0.855922** |
|  | **C32** | **0.663063** | **0.312269** | **0.606453** |
|  | **C33** | **0.677829** | **0.332849** | **0.856403** |
|  | **C34** | **0.605674** | **0.262452** | **0.855996** |
|  | **C35** | **0.707657** | **0.402762** | **0.606783** |
|  | **C36** | **0.700454** | **0.378553** | **0.856748** |
|  | **C37** | **0.713686** | **0.453424** | **0.106957** |
|  | **C38** | **0.711807** | **0.478657** | **0.356967** |
|  | **C39** | **0.688634** | **0.551061** | **0.106988** |
|  | **C40** | **0.699047** | **0.528002** | **0.356921** |
|  | **C41** | **0.579537** | **0.653463** | **0.106197** |
|  | **C42** | **0.660412** | **0.593527** | **0.106711** |
|  | **C43** | **0.643134** | **0.611933** | **0.356727** |
|  | **C44** | **0.530483** | **0.667255** | **0.106076** |
|  | **C45** | **0.505348** | **0.669831** | **0.356059** |
|  | **C46** | **0.602213** | **0.642359** | **0.356505** |
|  | **C47** | **0.429899** | **0.660742** | **0.106099** |
|  | **C48** | **0.454486** | **0.666507** | **0.355963** |
|  | **C49** | **0.310844** | **0.570292** | **0.10584** |
|  | **C50** | **0.383133** | **0.640461** | **0.106235** |
|  | **C51** | **0.362168** | **0.626339** | **0.35597** |
|  | **C52** | **0.28823** | **0.524608** | **0.10553** |
|  | **C53** | **0.281035** | **0.500384** | **0.355582** |
|  | **C54** | **0.325652** | **0.590791** | **0.355855** |
|  | **C55** | **0.276961** | **0.424528** | **0.105213** |
|  | **C56** | **0.27527** | **0.449724** | **0.355511** |
|  | **C57** | **0.345242** | **0.291091** | **0.105162** |
|  | **C58** | **0.289401** | **0.375068** | **0.105216** |
|  | **C59** | **0.299646** | **0.351964** | **0.35524** |
|  | **C60** | **0.386244** | **0.260746** | **0.105226** |
|  | **C61** | **0.408994** | **0.249711** | **0.355245** |
|  | **C62** | **0.327914** | **0.309525** | **0.355005** |
|  | **C63** | **0.48329** | **0.233402** | **0.10555** |
|  | **C64** | **0.458109** | **0.235957** | **0.355564** |
|  | **C65** | **0.626528** | **0.276713** | **0.106033** |
|  | **C66** | **0.534186** | **0.236558** | **0.105777** |
|  | **C67** | **0.558783** | **0.242373** | **0.356024** |
|  | **C68** | **0.663007** | **0.312332** | **0.106351** |
|  | **C69** | **0.677823** | **0.33284** | **0.356499** |
|  | **C70** | **0.605591** | **0.262623** | **0.356134** |
|  | **C71** | **0.707825** | **0.402737** | **0.106892** |
|  | **C72** | **0.700438** | **0.378545** | **0.356634** |
|  | **O1** | **0.432192** | **0.450774** | **0.248146** |
|  | **O2** | **0.464819** | **0.393717** | **0.749299** |
|  | **O3** | **0.529953** | **0.393634** | **0.2505** |
|  | **O4** | **0.464416** | **0.507364** | **0.749533** |
|  | **O5** | **0.52984** | **0.507726** | **0.250077** |
|  | **O6** | **0.562605** | **0.45097** | **0.751085** |
|  | **Zn1** | **0.419225** | **0.450418** | **0.845492** |
|  | **Zn2** | **0.458308** | **0.38283** | **0.346255** |
|  | **Zn3** | **0.53663** | **0.383123** | **0.847735** |
|  | **Zn4** | **0.458066** | **0.518667** | **0.346305** |
|  | **Zn5** | **0.536192** | **0.518594** | **0.847335** |
|  | **Zn6** | **0.574923** | **0.450725** | **0.34832** |

**Table S5**. The fully optimized cell parameters and atomic coordinates of ZnO@(10,10) CNT.

| **Cell parameters** | **Atom** | ***x*** | ***y*** | ***z*** |
| --- | --- | --- | --- | --- |
| **a = 31.334280 Å**  **b = 31.409719 Å**  **c = 4.932309 Å** | **H1** | **0.558423** | **0.559797** | **0.768653** |
|  | **H2** | **0.572554** | **0.583852** | **0.123893** |
|  | **H3** | **0.471631** | **0.559649** | **0.268098** |
|  | **H4** | **0.457423** | **0.583845** | **0.623112** |
|  | **H5** | **0.401735** | **0.484099** | **0.116914** |
|  | **H6** | **0.429156** | **0.484994** | **0.766421** |
|  | **H7** | **0.472351** | **0.409776** | **0.266462** |
|  | **H8** | **0.457119** | **0.386544** | **0.622578** |
|  | **H9** | **0.558705** | **0.410069** | **0.76904** |
|  | **H10** | **0.574033** | **0.386442** | **0.124035** |
|  | **H11** | **0.601395** | **0.485013** | **0.268857** |
|  | **H12** | **0.629257** | **0.484526** | **0.623319** |
|  | **C1** | **0.722772** | **0.547596** | **0.527403** |
|  | **C2** | **0.705435** | **0.589748** | **0.527314** |
|  | **C3** | **0.693556** | **0.608997** | **0.777263** |
|  | **C4** | **0.727964** | **0.525618** | **0.777376** |
|  | **C5** | **0.646176** | **0.657871** | **0.527077** |
|  | **C6** | **0.663599** | **0.643424** | **0.777214** |
|  | **C7** | **0.518939** | **0.701799** | **0.527173** |
|  | **C8** | **0.606923** | **0.68123** | **0.527208** |
|  | **C9** | **0.585946** | **0.689693** | **0.777271** |
|  | **C10** | **0.473473** | **0.698057** | **0.527392** |
|  | **C11** | **0.451534** | **0.692688** | **0.777389** |
|  | **C12** | **0.541485** | **0.700094** | **0.777176** |
|  | **C13** | **0.39048** | **0.662854** | **0.527523** |
|  | **C14** | **0.409606** | **0.674873** | **0.777384** |
|  | **C15** | **0.309854** | **0.555731** | **0.527692** |
|  | **C16** | **0.356251** | **0.63287** | **0.527567** |
|  | **C17** | **0.341797** | **0.615557** | **0.777554** |
|  | **C18** | **0.299404** | **0.511439** | **0.527684** |
|  | **C19** | **0.297747** | **0.488966** | **0.777814** |
|  | **C20** | **0.318342** | **0.576607** | **0.777657** |
|  | **C21** | **0.306731** | **0.421636** | **0.527821** |
|  | **C22** | **0.301466** | **0.443589** | **0.777863** |
|  | **C23** | **0.382694** | **0.31065** | **0.52825** |
|  | **C24** | **0.324141** | **0.37949** | **0.528018** |
|  | **C25** | **0.335909** | **0.360152** | **0.77804** |
|  | **C26** | **0.421741** | **0.28691** | **0.528369** |
|  | **C27** | **0.442753** | **0.278419** | **0.778329** |
|  | **C28** | **0.365498** | **0.325388** | **0.778127** |
|  | **C29** | **0.509877** | **0.266776** | **0.52842** |
|  | **C30** | **0.487278** | **0.268268** | **0.778429** |
|  | **C31** | **0.638386** | **0.306145** | **0.528007** |
|  | **C32** | **0.555331** | **0.270882** | **0.528286** |
|  | **C33** | **0.577264** | **0.276406** | **0.778286** |
|  | **C34** | **0.672672** | **0.336096** | **0.5279** |
|  | **C35** | **0.687107** | **0.353457** | **0.777911** |
|  | **C36** | **0.619231** | **0.294182** | **0.778133** |
|  | **C37** | **0.718959** | **0.413449** | **0.527714** |
|  | **C38** | **0.710443** | **0.39254** | **0.777729** |
|  | **C39** | **0.72952** | **0.457693** | **0.527573** |
|  | **C40** | **0.731342** | **0.480218** | **0.777429** |
|  | **C41** | **0.722769** | **0.547597** | **0.027317** |
|  | **C42** | **0.705431** | **0.589747** | **0.027311** |
|  | **C43** | **0.693559** | **0.609003** | **0.277278** |
|  | **C44** | **0.727917** | **0.52562** | **0.277429** |
|  | **C45** | **0.646197** | **0.657911** | **0.027179** |
|  | **C46** | **0.66361** | **0.643427** | **0.277132** |
|  | **C47** | **0.518938** | **0.701792** | **0.027192** |
|  | **C48** | **0.606948** | **0.681266** | **0.027308** |
|  | **C49** | **0.585946** | **0.689706** | **0.277207** |
|  | **C50** | **0.473472** | **0.698056** | **0.02735** |
|  | **C51** | **0.451544** | **0.692664** | **0.277435** |
|  | **C52** | **0.541493** | **0.700106** | **0.277143** |
|  | **C53** | **0.390509** | **0.662831** | **0.027397** |
|  | **C54** | **0.40961** | **0.674835** | **0.277473** |
|  | **C55** | **0.309834** | **0.555725** | **0.02772** |
|  | **C56** | **0.356265** | **0.632848** | **0.027523** |
|  | **C57** | **0.341793** | **0.615556** | **0.277608** |
|  | **C58** | **0.299382** | **0.511443** | **0.027789** |
|  | **C59** | **0.297709** | **0.488963** | **0.277737** |
|  | **C60** | **0.318341** | **0.576603** | **0.277657** |
|  | **C61** | **0.306729** | **0.421631** | **0.027905** |
|  | **C62** | **0.301436** | **0.443591** | **0.277815** |
|  | **C63** | **0.382712** | **0.310675** | **0.028183** |
|  | **C64** | **0.324136** | **0.37949** | **0.028005** |
|  | **C65** | **0.33591** | **0.360163** | **0.278061** |
|  | **C66** | **0.421766** | **0.286942** | **0.02828** |
|  | **C67** | **0.442754** | **0.278431** | **0.278397** |
|  | **C68** | **0.365504** | **0.325395** | **0.278199** |
|  | **C69** | **0.509884** | **0.266774** | **0.0284** |
|  | **C70** | **0.487284** | **0.268281** | **0.278429** |
|  | **C71** | **0.638409** | **0.306121** | **0.028126** |
|  | **C72** | **0.55533** | **0.27088** | **0.028353** |
|  | **C73** | **0.577279** | **0.276376** | **0.278224** |
|  | **C74** | **0.672693** | **0.336071** | **0.027969** |
|  | **C75** | **0.687108** | **0.353459** | **0.277859** |
|  | **C76** | **0.619243** | **0.294148** | **0.278071** |
|  | **C77** | **0.718936** | **0.413457** | **0.027691** |
|  | **C78** | **0.710445** | **0.392537** | **0.277744** |
|  | **C79** | **0.729496** | **0.457704** | **0.027463** |
|  | **C80** | **0.731292** | **0.480209** | **0.277506** |
|  | **O1** | **0.456892** | **0.485075** | **0.671619** |
|  | **O2** | **0.486411** | **0.433654** | **0.172494** |
|  | **O3** | **0.544579** | **0.433868** | **0.673802** |
|  | **O4** | **0.485791** | **0.535844** | **0.173159** |

**Table S6.** The fully optimized cell parameters and atomic coordinates of ZnO@(11,11) CNT.

| **Cell parameters** | **Atom** | ***x*** | ***y*** | ***z*** |
| --- | --- | --- | --- | --- |
| **a = 33.022522 Å**  **b = 33.022461 Å**  **c = 4.932670 Å** | **H1** | **0.54491** | **0.567446** | **0.343709** |
|  | **H2** | **0.558325** | **0.59034** | **0.698839** |
|  | **H3** | **0.462494** | **0.567287** | **0.843129** |
|  | **H4** | **0.448994** | **0.59034** | **0.198069** |
|  | **H5** | **0.396104** | **0.495373** | **0.691889** |
|  | **H6** | **0.422154** | **0.496246** | **0.341479** |
|  | **H7** | **0.46317** | **0.424633** | **0.841539** |
|  | **H8** | **0.448712** | **0.402519** | **0.197519** |
|  | **H9** | **0.545173** | **0.424925** | **0.344069** |
|  | **H10** | **0.559725** | **0.402416** | **0.698989** |
|  | **H11** | **0.58571** | **0.496256** | **0.843899** |
|  | **H12** | **0.612183** | **0.495796** | **0.198269** |
|  | **C1** | **0.730308** | **0.511531** | **0.592449** |
|  | **C2** | **0.728203** | **0.532809** | **0.842459** |
|  | **C3** | **0.709856** | **0.594662** | **0.592439** |
|  | **C4** | **0.71787** | **0.57483** | **0.842449** |
|  | **C5** | **0.625955** | **0.690926** | **0.592459** |
|  | **C6** | **0.688099** | **0.632061** | **0.592459** |
|  | **C7** | **0.674825** | **0.64882** | **0.842449** |
|  | **C8** | **0.587429** | **0.710626** | **0.592449** |
|  | **C9** | **0.567194** | **0.717549** | **0.842449** |
|  | **C10** | **0.64341** | **0.678572** | **0.842449** |
|  | **C11** | **0.503321** | **0.726549** | **0.592449** |
|  | **C12** | **0.524684** | **0.7256** | **0.842459** |
|  | **C13** | **0.380894** | **0.690212** | **0.592449** |
|  | **C14** | **0.460267** | **0.722293** | **0.592439** |
|  | **C15** | **0.439496** | **0.717173** | **0.842449** |
|  | **C16** | **0.346981** | **0.663363** | **0.592429** |
|  | **C17** | **0.332269** | **0.647824** | **0.842459** |
|  | **C18** | **0.399392** | **0.700968** | **0.842449** |
|  | **C19** | **0.297557** | **0.593459** | **0.592459** |
|  | **C20** | **0.307299** | **0.612511** | **0.842459** |
|  | **C21** | **0.279755** | **0.467011** | **0.592479** |
|  | **C22** | **0.283541** | **0.552537** | **0.592449** |
|  | **C23** | **0.279567** | **0.531513** | **0.842449** |
|  | **C24** | **0.290088** | **0.425009** | **0.592459** |
|  | **C25** | **0.298102** | **0.405178** | **0.842439** |
|  | **C26** | **0.27765** | **0.488299** | **0.842439** |
|  | **C27** | **0.333133** | **0.35101** | **0.592459** |
|  | **C28** | **0.319859** | **0.367779** | **0.842449** |
|  | **C29** | **0.440764** | **0.28229** | **0.592449** |
|  | **C30** | **0.364548** | **0.321258** | **0.592429** |
|  | **C31** | **0.382003** | **0.308904** | **0.842459** |
|  | **C32** | **0.483274** | **0.274239** | **0.592459** |
|  | **C33** | **0.504637** | **0.27329** | **0.842429** |
|  | **C34** | **0.420529** | **0.289204** | **0.842459** |
|  | **C35** | **0.568462** | **0.282656** | **0.592459** |
|  | **C36** | **0.547691** | **0.277546** | **0.842399** |
|  | **C37** | **0.675689** | **0.352006** | **0.592449** |
|  | **C38** | **0.608566** | **0.298862** | **0.592469** |
|  | **C39** | **0.627064** | **0.309628** | **0.842449** |
|  | **C40** | **0.70065** | **0.387319** | **0.592429** |
|  | **C41** | **0.710401** | **0.406371** | **0.842459** |
|  | **C42** | **0.660968** | **0.336467** | **0.842419** |
|  | **C43** | **0.728391** | **0.468317** | **0.592429** |
|  | **C44** | **0.724418** | **0.447292** | **0.842459** |
|  | **C45** | **0.730308** | **0.511522** | **0.092459** |
|  | **C46** | **0.728203** | **0.532809** | **0.342429** |
|  | **C47** | **0.709856** | **0.594652** | **0.092459** |
|  | **C48** | **0.71787** | **0.574821** | **0.342449** |
|  | **C49** | **0.625955** | **0.690916** | **0.092449** |
|  | **C50** | **0.688099** | **0.632051** | **0.092449** |
|  | **C51** | **0.674825** | **0.648811** | **0.342449** |
|  | **C52** | **0.587429** | **0.710616** | **0.092439** |
|  | **C53** | **0.567194** | **0.71754** | **0.342459** |
|  | **C54** | **0.64341** | **0.678563** | **0.342469** |
|  | **C55** | **0.503312** | **0.72654** | **0.092449** |
|  | **C56** | **0.524684** | **0.725591** | **0.342459** |
|  | **C57** | **0.380894** | **0.690202** | **0.092459** |
|  | **C58** | **0.460267** | **0.722284** | **0.092459** |
|  | **C59** | **0.439496** | **0.717164** | **0.342449** |
|  | **C60** | **0.34699** | **0.663353** | **0.092449** |
|  | **C61** | **0.332269** | **0.647815** | **0.342459** |
|  | **C62** | **0.399392** | **0.700959** | **0.342429** |
|  | **C63** | **0.297557** | **0.593459** | **0.092449** |
|  | **C64** | **0.307299** | **0.612502** | **0.342449** |
|  | **C65** | **0.279755** | **0.467011** | **0.092449** |
|  | **C66** | **0.283541** | **0.552537** | **0.092439** |
|  | **C67** | **0.279567** | **0.531513** | **0.342469** |
|  | **C68** | **0.290088** | **0.425** | **0.092459** |
|  | **C69** | **0.298102** | **0.405168** | **0.342459** |
|  | **C70** | **0.27765** | **0.488299** | **0.342439** |
|  | **C71** | **0.333133** | **0.351** | **0.092459** |
|  | **C72** | **0.319859** | **0.367769** | **0.342439** |
|  | **C73** | **0.440764** | **0.282281** | **0.092469** |
|  | **C74** | **0.364548** | **0.321248** | **0.092449** |
|  | **C75** | **0.382003** | **0.308895** | **0.342449** |
|  | **C76** | **0.483264** | **0.27423** | **0.092459** |
|  | **C77** | **0.504637** | **0.273281** | **0.342429** |
|  | **C78** | **0.420529** | **0.289195** | **0.342449** |
|  | **C79** | **0.568462** | **0.282647** | **0.092449** |
|  | **C80** | **0.547691** | **0.277537** | **0.342419** |
|  | **C81** | **0.675689** | **0.351996** | **0.092439** |
|  | **C82** | **0.608566** | **0.298862** | **0.092449** |
|  | **C83** | **0.627064** | **0.309618** | **0.342459** |
|  | **C84** | **0.70065** | **0.387319** | **0.092419** |
|  | **C85** | **0.710401** | **0.406361** | **0.342449** |
|  | **C86** | **0.660977** | **0.336467** | **0.342429** |
|  | **C87** | **0.728391** | **0.468308** | **0.092459** |
|  | **C88** | **0.724418** | **0.447283** | **0.342459** |
|  | **O1** | **0.448496** | **0.496322** | **0.246579** |
|  | **O2** | **0.476529** | **0.447368** | **0.747449** |
|  | **O3** | **0.531768** | **0.447574** | **0.248769** |
|  | **O4** | **0.475937** | **0.544637** | **0.748129** |
|  | **O5** | **0.531655** | **0.544712** | **0.248919** |
|  | **O6** | **0.559359** | **0.496284** | **0.749249** |
|  | **Zn1** | **0.437495** | **0.495927** | **0.843289** |
|  | **Zn2** | **0.470554** | **0.438114** | **0.343419** |
|  | **Zn3** | **0.537752** | **0.438039** | **0.844869** |
|  | **Zn4** | **0.470328** | **0.554332** | **0.344159** |
|  | **Zn5** | **0.537188** | **0.554332** | **0.844889** |
|  | **Zn6** | **0.570463** | **0.496143** | **0.345439** |

**Table S7**. The fully optimized cell parameters and atomic coordinates of ZnO@(12,12) CNT.

| **Cell parameters** | **Atom** | ***x*** | ***y*** | ***z*** |
| --- | --- | --- | --- | --- |
| **a = 35.022980 Å**  **b = 35.022499 Å**  **c = 4.933630 Å** | **H1** | **0.557527** | **0.554438** | **0.343717** |
|  | **H2** | **0.570182** | **0.576017** | **0.698837** |
|  | **H3** | **0.479817** | **0.554283** | **0.843137** |
|  | **H4** | **0.467099** | **0.576017** | **0.198077** |
|  | **H5** | **0.417231** | **0.486485** | **0.691897** |
|  | **H6** | **0.44179** | **0.487298** | **0.341487** |
|  | **H7** | **0.480458** | **0.419774** | **0.841547** |
|  | **H8** | **0.466825** | **0.398927** | **0.197527** |
|  | **H9** | **0.557774** | **0.420058** | **0.344077** |
|  | **H10** | **0.571499** | **0.398827** | **0.698997** |
|  | **H11** | **0.596003** | **0.487308** | **0.843907** |
|  | **H12** | **0.620955** | **0.486878** | **0.198277** |
|  | **C1** | **0.747363** | **0.490809** | **0.598537** |
|  | **C2** | **0.713468** | **0.60662** | **0.598547** |
|  | **C3** | **0.742865** | **0.531361** | **0.598557** |
|  | **C4** | **0.738037** | **0.550946** | **0.848557** |
|  | **C5** | **0.689293** | **0.63949** | **0.598577** |
|  | **C6** | **0.675331** | **0.654028** | **0.848557** |
|  | **C7** | **0.723197** | **0.588946** | **0.848557** |
|  | **C8** | **0.626213** | **0.689971** | **0.598557** |
|  | **C9** | **0.643466** | **0.67952** | **0.848557** |
|  | **C10** | **0.508967** | **0.718517** | **0.598557** |
|  | **C11** | **0.588844** | **0.706347** | **0.598567** |
|  | **C12** | **0.569469** | **0.711952** | **0.848557** |
|  | **C13** | **0.468425** | **0.714018** | **0.598547** |
|  | **C14** | **0.44884** | **0.709191** | **0.848557** |
|  | **C15** | **0.529137** | **0.718105** | **0.848557** |
|  | **C16** | **0.393165** | **0.684622** | **0.598557** |
|  | **C17** | **0.41084** | **0.694351** | **0.848547** |
|  | **C18** | **0.309823** | **0.597367** | **0.598557** |
|  | **C19** | **0.360304** | **0.660456** | **0.598557** |
|  | **C20** | **0.345748** | **0.646476** | **0.848547** |
|  | **C21** | **0.293447** | **0.560007** | **0.598537** |
|  | **C22** | **0.287833** | **0.540623** | **0.848557** |
|  | **C23** | **0.320265** | **0.61463** | **0.848557** |
|  | **C24** | **0.281268** | **0.48013** | **0.598567** |
|  | **C25** | **0.28168** | **0.500291** | **0.848557** |
|  | **C26** | **0.315163** | **0.364329** | **0.598557** |
|  | **C27** | **0.285767** | **0.439579** | **0.598557** |
|  | **C28** | **0.290594** | **0.419994** | **0.848557** |
|  | **C29** | **0.339338** | **0.331458** | **0.598547** |
|  | **C30** | **0.353309** | **0.316902** | **0.848557** |
|  | **C31** | **0.305434** | **0.381994** | **0.848557** |
|  | **C32** | **0.402418** | **0.280977** | **0.598557** |
|  | **C33** | **0.385165** | **0.29141** | **0.848557** |
|  | **C34** | **0.519655** | **0.252432** | **0.598557** |
|  | **C35** | **0.439788** | **0.264602** | **0.598557** |
|  | **C36** | **0.459162** | **0.258978** | **0.848557** |
|  | **C37** | **0.560206** | **0.25693** | **0.598547** |
|  | **C38** | **0.579801** | **0.261749** | **0.848557** |
|  | **C39** | **0.499494** | **0.252834** | **0.848557** |
|  | **C40** | **0.635466** | **0.286326** | **0.598547** |
|  | **C41** | **0.617792** | **0.276589** | **0.848557** |
|  | **C42** | **0.718808** | **0.373582** | **0.598547** |
|  | **C43** | **0.668327** | **0.310492** | **0.598567** |
|  | **C44** | **0.682884** | **0.324463** | **0.848557** |
|  | **C45** | **0.735184** | **0.410933** | **0.598577** |
|  | **C46** | **0.740798** | **0.430308** | **0.848557** |
|  | **C47** | **0.708366** | **0.35631** | **0.848547** |
|  | **C48** | **0.746952** | **0.470639** | **0.848547** |
|  | **C49** | **0.747363** | **0.4908** | **0.098557** |
|  | **C50** | **0.713468** | **0.606611** | **0.098557** |
|  | **C51** | **0.742865** | **0.531351** | **0.098547** |
|  | **C52** | **0.738037** | **0.550937** | **0.348547** |
|  | **C53** | **0.689293** | **0.639481** | **0.098567** |
|  | **C54** | **0.675331** | **0.654028** | **0.348547** |
|  | **C55** | **0.723197** | **0.588937** | **0.348557** |
|  | **C56** | **0.626213** | **0.689962** | **0.098557** |
|  | **C57** | **0.643466** | **0.679511** | **0.348567** |
|  | **C58** | **0.508967** | **0.718508** | **0.098567** |
|  | **C59** | **0.588844** | **0.706338** | **0.098567** |
|  | **C60** | **0.569469** | **0.711943** | **0.348557** |
|  | **C61** | **0.468425** | **0.714** | **0.098557** |
|  | **C62** | **0.44884** | **0.709182** | **0.348557** |
|  | **C63** | **0.529137** | **0.718096** | **0.348557** |
|  | **C64** | **0.393165** | **0.684613** | **0.098557** |
|  | **C65** | **0.41084** | **0.694342** | **0.348537** |
|  | **C66** | **0.309823** | **0.597358** | **0.098547** |
|  | **C67** | **0.360304** | **0.660447** | **0.098567** |
|  | **C68** | **0.345748** | **0.646467** | **0.348557** |
|  | **C69** | **0.293447** | **0.559998** | **0.098547** |
|  | **C70** | **0.287833** | **0.540623** | **0.348567** |
|  | **C71** | **0.320265** | **0.61462** | **0.348557** |
|  | **C72** | **0.281268** | **0.480121** | **0.098557** |
|  | **C73** | **0.28168** | **0.500291** | **0.348557** |
|  | **C74** | **0.315163** | **0.36432** | **0.098547** |
|  | **C75** | **0.285767** | **0.43957** | **0.098557** |
|  | **C76** | **0.290585** | **0.419985** | **0.348577** |
|  | **C77** | **0.339338** | **0.331449** | **0.098557** |
|  | **C78** | **0.353309** | **0.316893** | **0.348567** |
|  | **C79** | **0.305434** | **0.381985** | **0.348547** |
|  | **C80** | **0.402418** | **0.280968** | **0.098557** |
|  | **C81** | **0.385156** | **0.291401** | **0.348557** |
|  | **C82** | **0.519655** | **0.252423** | **0.098557** |
|  | **C83** | **0.439788** | **0.264592** | **0.098567** |
|  | **C84** | **0.459162** | **0.258969** | **0.348557** |
|  | **C85** | **0.560206** | **0.256921** | **0.098547** |
|  | **C86** | **0.579801** | **0.26174** | **0.348557** |
|  | **C87** | **0.499494** | **0.252825** | **0.348557** |
|  | **C88** | **0.635466** | **0.286317** | **0.098547** |
|  | **C89** | **0.617792** | **0.276579** | **0.348567** |
|  | **C90** | **0.718808** | **0.373573** | **0.098557** |
|  | **C91** | **0.668327** | **0.310483** | **0.098557** |
|  | **C92** | **0.682884** | **0.324454** | **0.348557** |
|  | **C93** | **0.735184** | **0.410933** | **0.098567** |
|  | **C94** | **0.740798** | **0.430308** | **0.348547** |
|  | **C95** | **0.708366** | **0.356301** | **0.348557** |
|  | **C96** | **0.746952** | **0.47063** | **0.348547** |
|  | **O1** | **0.466633** | **0.487372** | **0.246567** |
|  | **O2** | **0.493057** | **0.441225** | **0.747437** |
|  | **O3** | **0.545129** | **0.441417** | **0.248757** |
|  | **O4** | **0.492499** | **0.532924** | **0.748107** |
|  | **O5** | **0.545028** | **0.532997** | **0.248917** |
|  | **O6** | **0.571151** | **0.487335** | **0.749237** |
|  | **Zn1** | **0.456255** | **0.486997** | **0.843287** |
|  | **Zn2** | **0.487416** | **0.432493** | **0.343427** |
|  | **Zn3** | **0.55078** | **0.43242** | **0.844867** |
|  | **Zn4** | **0.487205** | **0.542067** | **0.344167** |
|  | **Zn5** | **0.550249** | **0.542067** | **0.844897** |
|  | **Zn6** | **0.58162** | **0.487198** | **0.345447** |

**Table S8.** Total and HOMO, LUMO energies of ZnO@(9,9) CNT under different uniaxial strains. The HOMO and LUMO energies are taken the Fermi energy (0 eV) as reference.

| **Uniaxial Strain** | **Total energy (eV)** | **HOMO energy (eV)** | **LUMO energy (eV)** |
| --- | --- | --- | --- |
| **-15%** | **-696.58127003** | **-0.4224** | **0.5938** |
| **-9%** | **-731.47252259** | **-0.8276** | **0.8043** |
| **-7%** | **-739.87404714** | **-0.7850** | **0.8481** |
| **-5%** | **-745.97888053** | **-0.7894** | **0.8409** |
| **-3%** | **-749.90192977** | **-0.7866** | **0.8480** |
| **0%** | **-752.17149564** | **-0.7898** | **0.8493** |
| **3%** | **-750.65195316** | **-0.7022** | **0.8284** |
| **5%** | **-747.80246713** | **-0.5910** | **0.6652** |
| **7%** | **-743.68847134** | **-0.4369** | **0.5299** |
| **9%** | **-738.46773774** | **-0.2425** | **0.4261** |
| **15%** | **-718.14438438** | **-0.1560** | **0.3185** |
